# Glycogen Synthase Kinase-3 Inhibitors: Preclinical and Clinical Focus on CNS-A Decade Onward

**DOI:** 10.3389/fnmol.2021.792364

**Published:** 2022-01-21

**Authors:** Sara Melisa Arciniegas Ruiz, Hagit Eldar-Finkelman

**Affiliations:** The Department of Human Molecular Genetics and Biochemistry Sackler School of Medicine, Tel Aviv University, Tel Aviv, Israel

**Keywords:** GSK-3, inhibitors, CNS, neurodegeneration, drug development

## Abstract

The protein kinase, GSK-3, participates in diverse biological processes and is now recognized a promising drug discovery target in treating multiple pathological conditions. Over the last decade, a range of newly developed GSK-3 inhibitors of diverse chemotypes and inhibition modes has been developed. Even more conspicuous is the dramatic increase in the indications that were tested from mood and behavior disorders, autism and cognitive disabilities, to neurodegeneration, brain injury and pain. Indeed, clinical and pre-clinical studies were largely expanded uncovering new mechanisms and novel insights into the contribution of GSK-3 to neurodegeneration and central nerve system (CNS)-related disorders. In this review we summarize new developments in the field and describe the use of GSK-3 inhibitors in the variety of CNS disorders. This remarkable volume of information being generated undoubtedly reflects the great interest, as well as the intense hope, in developing potent and safe GSK-3 inhibitors in clinical practice.

## Introduction

Glycogen synthase kinase-3 (GSK-3) is a highly conserved protein serine/threonine kinase that plays a central role in a wide variety of cellular processes concerned with coordinating catabolic and anabolic pathways (Eldar-Finkelman, 2002; Ying et al., 2008; Hur and Zhou, 2010; Beurel et al., 2015; Cormier and Woodgett, 2017). Glycogen synthase kinase-3 targeted phosphorylation typically inhibits the activity of the substrate, leading to attenuation of the signaling pathway. Hence, active GSK-3 maintains cellular homeostasis, while its inhibition stimulates biological responses. Particular interest has been focused on GSK-3 in the context of neuronal and brain functions, while GSK-3 activity is essential for normal neurodevelopment, GSK-3 hyperactivity is implicated in psychiatric conditions, cognitive dysfunction, and neurodegeneration (Zhou and Snider, 2005; Kim et al., 2009; Eldar-Finkelman and Martinez, 2011; Llorens-Martin et al., 2014; Beurel et al., 2015). The current paradigm thus suggests that GSK-3 hyperactivity is a causative factor in disease pathogenesis, while inhibition of GSK-3 is a potential therapeutic avenue (Eldar-Finkelman and Martinez, 2011; Kaidanovich-Beilin et al., 2012; King et al., 2014; Pandey and DeGrado, 2016; Sayas and Avila, 2021).

Glycogen synthase kinase-3 (GSK-3) exists as two isozymes that are encoded by two separate genes, GSK-3α and β (Woodgett, 1990). A splice variant of GSK-3β with a 13-residue insert in the catalytic domain has also been described (Mukai et al., 2002). The GSK-3 isozymes share 98% identity in the catalytic domains, but there are significantly differences in the N- and C-terminal domains. From the evolutionary perspective, the α and β isozymes diverged from a common precursor around the time vertebrates emerged, and both genes are highly conserved in fish, amphibians, reptiles, and mammals (Alon et al., 2011). The GSK-3 isozymes exhibit both overlapping and distinct functions in the nervous systems. In some cases, there may be an absolute requirement for a given isozyme for a certain process, but in other cases, the activities of the two isoforms may be completely interchangeable (Doble et al., 2007; Force and Woodgett, 2009; Rippin and Eldar-Finkelman, 2021). An absolute need for a certain isozyme is probably dependent on dosage, targeting, substrate, and timing.

It is not surprising that tremendous efforts have been invested in developing GSK-3 inhibitors as potential drugs for treating neurodegenerative and psychiatric disorders (as well as other indications not described here). Over the past two decades, a large number of highly diverse classes of GSK-3 inhibitors have been developed by pharmaceutical companies, and academic institutions. In our previous review (Eldar-Finkelman and Martinez, 2011), we categorized GSK-3 inhibitors into four classes: (*i).* cations that include the mood stabilizer lithium (e.g., lithium salts) (Snitow et al., 2021), and additional metal ions such as zinc and copper that inhibit GSK-3 in the millimolar or sub-micromolar ranges, respectively (Klein and Melton, 1996; Ilouz et al., 2002). (*ii).* ATP competitive inhibitors as represented by small molecules that target the ATP binding site of the kinase. These may either be synthetic organic molecules or derived from natural sources. (*iii).* Allosteric non-ATP competitive inhibitors, or *(iv)*. Substrate competitive inhibitors (SCIs) comprising peptides or molecules that target the substrate binding site of the kinase. An updated search clearly indicates that the field is “exploding” with a growing number of new scaffolds and molecules discovered to be potent GSK-3 inhibitors. This is thanks to the emergence of new computational tools that enable precise molecular modeling and screening of a huge number of molecules. The majority of the newly developed/discovered GSK-3 inhibitors fall into the second class, namely the ATP competitive inhibitors. These, however, are characterized by safety issues and low specificity, and they also tend to induce drug resistance (due to the formation of point mutations in the ATP binding site of the kinase). Although their discovery is more challenging, compounds that recognize other regions of the kinase are considered a favorable choice as the target is more conserved (Avrahami et al., 2013).

The literature reflects the significant progress made since 2011. First, and maybe most importantly, some GSK-3 inhibitors have reached the clinic. These products include AZD1080 from AstraZeneca and LY2090314 from Eli-Lilly (both ATP competitive inhibitors). Unfortunately, their development was discontinued due to safety issues (Rizzieri et al., 2016; Bhat et al., 2018). In addition, an allosteric non-reversible ATP competitive inhibitor, Tideglusib (also known as NP12, NP031112) (Martinez et al., 2002), developed by Noscira, reached Phase II trials. No safety issues were reported, but the drug did not reach the primary end points in patients with Alzheimer’s disease (AD) or Progressive Supranuclear Palsy (PSP) (Tolosa et al., 2014; Lovestone et al., 2015). However, clinical trials with Tideglusib have also been conducted in myotonic dystrophy NCT02858908, NCT03692312, and autism (Anagnostou, 2018), and its use in tooth repair is also being investigated. Second, GSK-3-isoform specific inhibitors have been developed. These include oxadiazole-based selective GSK3α inhibitors (Lo Monte et al., 2012; Neumann et al., 2015), and two inhibitors termed BRD0705 and BRD3731, which are reported to be selective for GSK-3α and GSK-3β, respectively. The isozyme specificity is based on a single “switch” in the hinge binding domain (Wagner et al., 2018). Third, is the development of small molecules SCIs that, as already described, represent a favorable mode of inhibition. These include 5-iminothiadiazoles (ITDZ compounds) and isoorientin analogs (Palomo et al., 2012; Liang and Li, 2018). Selective potent small molecule SCIs were developed for GSK-3 SCI peptides based on our binding model (Rippin et al., 2020).

A major problem in central nerve system (CNS) therapy is the challenge of penetration of drugs into the brain. The use of positron emission tomography (PET) imaging was used to trace the entry of GSK-3 inhibitors into the brain and provided additional tools in developing effective GSK-3 related CNS penetrating drugs (Pandey and DeGrado, 2016).

Taken together, the remarkable increase in the number of GSK-3 inhibitors developed, undoubtedly reflects the great interest in, as well as the hope of, identifying GSK-3 inhibitors suitable for clinical use. To date, none of the GSK-3 inhibitors tested has reached the market.

A detailed description of the variety of GSK-3 inhibitors developed can be found in a collection of reviews (Eldar-Finkelman and Martinez, 2011; Pandey and DeGrado, 2016; Palomo and Martinez, 2017; Saraswati et al., 2018; Roca and Campillo, 2020). In this section, we summarize the reported GSK-3 inhibitors by their different chemical scaffolds or source. The compounds derived from natural sources come mainly from plants or marine organisms, and include Indirubin, Hymenialdisine, Meridianins, Manzamines, Palinurin, and Tricantin (Eldar-Finkelman and Martinez, 2011; Pandey and DeGrado, 2016). Also worth mentioning in this context, is BIO (6- bromoindirubin-3-oxime), a synthetic indirubin that has been widely used as a GSK-3 inhibitor (Leclerc et al., 2001). Most of the reported inhibitors are synthetic organic molecules that function as ATP competitive inhibitors. These include **Maleimides,** of which the first examples are the bisarylmaleimide compounds SB-216763 and SB-415286 developed by GlaxoSmithKline (Coghlan et al., 2000). Subsequently, numerous maleimide derivates with improved selectivity and pharmacological properties have been synthesized. These include BIP-135, indolylmaleimides, heteroaryl-maleimides, and macrocyclic azaindolylmaleimides, among others (Pandey and DeGrado, 2016; Saraswati et al., 2018). The first **Pyridine and Pyrimidine** compounds were the aminopyrimidines CT98014, CT98023 and CT99021 developed by Chiron (Ring et al., 2003), although additional derivates reported to be potent GSK-3 inhibitors include pyrazolopyrimidines, pyrazolopyridines, imidazopyridine, the pyrrolopyrimidine TWS119, the oxindolypyridine AZD1080 from AstraZeneca, SAR502250 from Sanofi, and the triazolopyridine JGK-263 (Peat et al., 2004; Bhat et al., 2018; Saraswati et al., 2018; Griebel et al., 2019; Buonfiglio et al., 2020). In addition, **Pyridinones**, **Benzimidazoles**, and **Indazole** derivatives including 7-hydroxy-1H-benzoimidazole (Shin D. et al., 2007; Coffman et al., 2011), and a 1H-indazole-3-carboxamide core compound termed AF3581 (Furlotti et al., 2015), have been reported. The **Thiazole** group was initially represented by the amino thiazoles AR-A014418 from AstraZeneca (Bhat et al., 2018), and later derivatives such as VP2.51 and VP2.54 have followed (Morales-Garcia et al., 2012; de Munck et al., 2016). **Paullone** compounds include Kenpaullone, Alsterpaullone and Cazpaullone (Kunick et al., 2004; Stukenbrock et al., 2008). **Pyrazine** analogs have been developed (Berg et al., 2012), and the addition of a halomethylketone moiety was found to result in a more potent irreversible inhibitor (Perez et al., 2011). Notably, a pyrazine analog termed AZD2858 developed by AstraZeneca demonstrated good efficacy but was discontinued due to toxic side effects (Bhat et al., 2018). 1**,3,4-oxadiazole derivates** MMBO and TCS2002 (Onishi et al., 2011) have been reported to demonstrate selectivity toward GSK-3α (Lo Monte et al., 2012; Neumann et al., 2015). **Pyridinyl isonicotinamides** developed by Bristol Myers Co (Luo et al., 2016) have a unique mode of binding to the ATP binding site in which the ‘left-side’ of the molecule (aminopyridine) is bound to the hinge region while the ‘right-side’ of the molecule (amide carbonyl) forms a hydrogen bond with Lys-85, a critical residue for ATP binding (Luo et al., 2016). Other examples include the **Oxazole-carboxamide** derivates such as PF-04802367 (PF-367) developed by Pfizer (Liang et al., 2016). The **Pyrazole** or **Triazole** scaffolds from Teijin Pharma Vertex and Abott Astex Therapeutics (Palomo and Martinez, 2017). The selective GSK-3 isozyme inhibitors, BRD0705 and BRD3731, belong to the family of **Pyrazolodihydropyridines** (Wagner et al., 2016).

**Thiadiazolidinones** belong to the class of non-ATP competitive and include the small heterocyclic TDZD inhibitor family and its clinically approved derivative, Tideglusib (NP031112, NP-12) (Martinez et al., 2002; Palomo and Martinez, 2017). Additional new compounds, including the quinolone analogs VP0.7, VP3.35, and SC100, have been synthesized based on the heterocyclic thiadiazolidinone core (Morales-Garcia et al., 2013; Arfeen et al., 2016; Palomo and Martinez, 2017), and two new classes of Chloromethyl thienyl ketones and Halomethyl phenyl ketones are now also reported as non-ATP competitive inhibitors (Conde et al., 2003).

A few molecules functioning as SCIs have now been described. The first GSK-3 SCI peptides developed included the potent L803mts and L807mts inhibitors that demonstrated high selectivity and good bioavailability (Plotkin et al., 2003; Licht-Murava et al., 2016). Small molecules with an **Anthracenone–isoxazole** core were discovered by using GSK-3-peptide binding models. These were shown to interact with the phosphate binding pocket and the substrate binding loop of the kinase (Rippin et al., 2020). **Iminothiadiazole** compounds (ITDZs) such as 5-imino-1,2,4-thiadiazoles, VP 1.14, and VP 1.16 have also been developed as GSK-3 SCIs (Palomo et al., 2012), and isoorientin analogs (Liang and Li, 2018). The GSK-3 inhibitors are also listed in Table 1, and those used in *in vivo* disease models are summarized in Table 2.

**TABLE 1:** GSK-3 inhibitors.

Type/class	Compound
**ATP competitive**	
Indoles	BIO, indirubin-3**’**-oxime
	
Maleimides	SB-216763, SB-415286
	BIP-135
Pyridines and pyrimidines	CHIR98014, CHIR98023, CHIR99021
	AZD1080, SAR502250
	IMID1, IMID2
	TWS119
	AZD1080, SAR502250
	JGK-263
Thiazole	AR-A014418
	VP2.51, VP2.54
Paullones	Kenpaullone, Alsterpaullone, Cazpaullone, Azakenpaullone
Pyrazine	AZD2858
Oxadiazole	MMBO, TCS2002
Isonicotinamides	Compounds 19, 25, and 33
Oxazole-carboxamide	PF-04802367 (PF-367)
Pyrazolodihydropyridine	BRD0705, BRD3731
indazole-carboxamide	AF3581
**Non-ATP competitive**	
Thiadiazolidinones	TDZD-8, Tideglusib (NP031112, NP-12)
	VP0.7, VP3.35, SC100
Chloromethyl thienyl and halomethyl phenyl ketones	GSK-3β Inhibitor VI
**Substrate competitive**	
Peptides	L803mt, L807mts
Anthracenone–isoxazole	4-1, 4-2, 4-3, 4-4, 4-5
Iminothiadiazoles	5-imino-1,2,4-thiadiazoles, VP 1.14, VP 1.16

*Inhibitors are sorted by mode of inhibition and chemical cores.*

**TABLE 2:** Use of GSK-3 inhibitors in clinical and pre-clinical experiments.

Disease	Clinical trials	GSK3 inhibitors used in in vivo models
Bipolar and mood disorders	Lithium (5)	Alsterpaullone, indirubins, AF3581, AR-A014418, L803mts, SB-627772, SB-216763, indolylmaleimides, TDZD-8, tideglusib, VP2.51
Schizophrenia		SB-216763, TDZD-8, BRD3731
Autism spectrum disorder	Lithium (9)	tideglusib
Fragile X syndrome	Lithium (3), Tideglusib (1)	AR-A014418, BRD0705, Chir99021, L803mts, TDZD-8, VP0.7, SB-216763, SB-415286
Rett syndrome	Lithium (1)	BIO, SB-216763
Phelan-Mc Dermid syndrome	–	Tideglusib
CDKL5 deficiency disorder	Lithium (2)	SB-216763, tideglusib
Intellectual disability	–	–
Conduct disorder	Lithium (1)	–
Spinocerebellar ataxia	Lithium (5)	–
Down syndrome	Lithium (1)	Tideglusib
Alzheimer’s	Lithium (1)	AR-A014418,, AZD2858, AZD1080,, isonicotinamides, isoorientin, L803mts, L807mts, MMBO, SAR502250, SB-216763, Indirubin-3**’**-monoxime, PF-04802367, TCS2002, tideglusib, C7a, C7b
Mild cognitive impairment	Lithium (6), Tideglusib (2)	
Parkinson	AZD1080 (2)	Alsterpaullone, AR-A014418, indirubin-3′-monoxime, SB-216763, SC001, TDZD-8, TWS119
Progressive supranuclear palsy	Lithium (7)	
Huntington’s disease	Lithium (1)	L807mts
Amyotrophic lateral sclerosis	Tideglusib (3)	AR-A014418, JGK-263, VP2.51
Multiple sclerosis	Lithium (4)	AR-A014418, indirubin, L803mts, TDZD-8, VP2.51, VP2.7, VP3.15, VP1.15
Cerebral hemorrhage	Lithium (5)	AR-A014418, BIO, TWS119
Brain ischemia	–	AR-A014418, BIO, CT025, SB-216763, TDZD-8, TIBPO, tideglusib, TWS119
Traumatic brain injury	–	CT99021, L803mts, SB-216763
Spinal cord injury	–	Ro3303544, SB-415286, TDZD-8
Neuropathic pain	Lithium (1)	AR-A014418, SB-216763, TZDZ-8
Epilepsy	Lithium (9)	Bio-acetoxime, indirubin, TCS2002, TDZD-8

*Clinical trials with lithium or GSK-3 inhibitors are indicate together with the number of studies conducted (in prentices). The GSK-3 inhibitors used in animal disease models are listed.*

**FIGURE 1:**
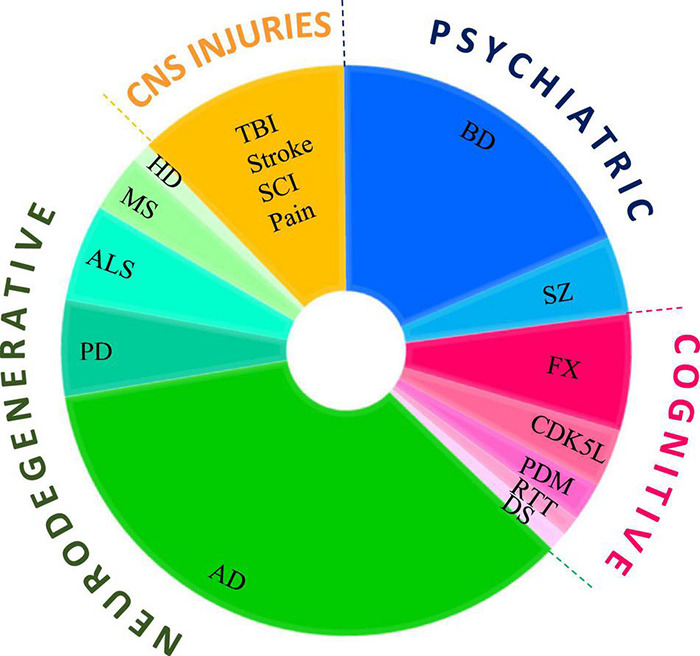
Use of GSK-3 inhibitors in CNS diseases models. Pie chart represents the number of studies that tested GSK-3 inhibitors in each disease category, psychiatry disorders, cognitive dysfunction, neurodegenerative disorders, CNS injuries and pain. Diseases abbreviations are found in the text.

In this review, we summarize the use of GSK-3 inhibitors in CNS disorders. It is important to reiterate that despite the intense activity in the field, only a few GSK-3 inhibitors have reached the clinic. Therefore, here we describe only the inhibitors used in *in vivo* models or in clinical trials. Although lithium is not a selective GSK-3 inhibitor, we have included clinical and pre-clinical studies with lithium, as we believe that they contribute to our understanding of the pathological role of GSK-3. In contrast, although valproic acid (VP), an approved antiepileptic drug, has been shown to inhibit GSK-3, *albeit* indirectly, and has been used in a number of clinical trials, we do not include studies with VP. Our approach is to first describe clinical trials (when available), and then discuss the potential role of GSK-3 in the specific disease, followed by studies of the GSK-3 inhibitors in relevant animal models of disease. Segmentation of the number of studies using GSK-3 inhibitors by diseases type is presented in Figure 1. It is clear that most of the studies were focused on the neurodegenerative arena, and particularly to AD.

## Psychiatric Disorders

### Bipolar and Mood Disorders

Bipolar disorder (BD) is a chronic neuropsychiatric condition that is characterized by cycles of manic and depressive episodes and affects 1–3% of the population worldwide. Common treatment regimens involve so-called “Mood stabilizers” of which Lithium salts (Lithium) are the favorite choice (Kessing, 2019). Lithium was first recommended for use as an anticonvulsant and hypnotic by the neurologist Silas Weir Mitchell in 1870, and was used in Demark in the late 19^th^ century as a treatment for prophylaxis of depression (Shorter, 2009). Lithium treatment was subsequently banned due to severe side effects (Shorter, 2009) but made a “come back” in 1949 with a new report of the efficacy of lithium for treating BD (Cade, 1999). In 1970, the United States approved the use of lithium, although it is well acknowledged that the enthusiasm for lithium as a therapeutic agent is tempered by the toxicity that occurs just beyond the narrow therapeutic range (van der Loos et al., 2009; Haussmann et al., 2015).

It is most likely that BD is caused by an interplay between genetic, epigenetic, and environmental factors. Nevertheless, the initial finding that lithium inhibits GSK-3 (Klein and Melton, 1996) made GSK-3 an important *in vivo* target for treating BD (Snitow et al., 2021). Indeed, GSK-3 inhibition replicates many of the effects of lithium on cellular and biological systems. Furthermore, genetic-based studies verified the contribution of abnormal GSK-3 to mood behavior: heterozygous GSK-3β ^(±)^ mice exhibit behavior that mimics chronic lithium treatment, and knockout GSK-3α^(–/–)^ mice display anti-depressive like activity (O’Brien et al., 2004; Kaidanovich-Beilin et al., 2009). Subsequent studies with, transgenic mice overexpressing GSK-3β indicated that the animals recapitulate manic behavior (Prickaerts et al., 2006), while constitutively active GSK-3 in knock-in mice increases the vulnerability to stress-induced depressive behavior (Polter et al., 2010). Studies in human patients suggested that GSK-3 may be a risk gene in BD, since increased protein levels of GSK-3α/β and increased GSK-3β activity could be detected in peripheral blood cells of BD patients (Li et al., 2010; Munkholm et al., 2018). Similarly, elevated GSK-3β mRNA levels were measured in the hippocampus of patients with major depression (Oh et al., 2010), and polymorphisms in the GSK-3β gene were reported in individuals with BD and major depression (Saus et al., 2011; Terao et al., 2020; Yang et al., 2020), and could be correlated with a therapeutic response to lithium (Benedetti et al., 2005). In this context, a GSK-3 polymorphism associated with insomnia in depressed patients (Costemale-Lacoste et al., 2018), also correlates with sleep disturbance in BD patients (Lamont et al., 2010). Finally, modulation of the serotonin neurotransmission system by GSK-3 is strongly implicated in BD (Polter and Li, 2011), and the interaction of GSK-3 with BD risk genes may be involved in the condition. For example, GSK-3 interacts with Disrupted-in-Schizophrenia 1 (DISC1), a candidate risk gene in affective disorders (Lipina et al., 2011). In addition, aberrant GSK-3 activity is reflected by activation of Akt (upstream regulator of GSK-3) due to an excess of dopamine (Beaulieu et al., 2009). Finally, GSK-3 may be activated by the BD susceptibility gene, *Trpm2* (Ca^2+^-permeable cation channel) (Jang et al., 2015), and serotonin deficiency (Beaulieu et al., 2008).

A large number of GSK-3 inhibitors have been tested in BD/depressive behavior models. In the mania model of amphetamine-induced hyperactivity in mice, treatment with a variety of GSK-3 inhibitors reduced the “manic” effects as manifested by reductions in hyperactivity, and normalization of ambulation, and stereotypic behavior (Kozikowski et al., 2007; Kalinichev and Dawson, 2011; Enman and Unterwald, 2012; Furlotti et al., 2015; Capurro et al., 2020; Prati et al., 2020). The compounds used in these studies included indirubins, Alsterpaullone, TDZD-8, AR-A014418, SB-216763, SB-627772, 3-benzofuranyl-4-indolylmaleimides, and AF3581. As already described, the potential impact of GSK-3 on the biological clock may be connected to the sleep disturbances suffered by BD patients (Lamont et al., 2010). Glycogen synthase kinase-3 has been suggested to disrupt circadian rhythmicity by regulating central circadian genes such as *Clock*, *Period*, and *Timeless* (Lamont et al., 2010; Paul et al., 2012). Accordingly, treatment with the indolyl maleimide, benzofuran-3-yl-(indol-3-yl), attenuated locomotor hyperactivity in *Clock* mutant mice (Kozikowski et al., 2011), and treatment with Kenpaullone lengthened the transcription period of the *Period 2* gene (Kaladchibachi et al., 2007). This delay was also observed in cells of BD patients treated with lithium (McCarthy et al., 2013). These findings may explain, at least in part, the long time period required for clinical effectiveness of lithium, because it reflects the gradual realignment of the circadian clock in patients.

Although lithium is mainly effective in treating the manic phase, the GSK-3 inhibitors also demonstrated significant benefits in treating the depressive phase (in animals). Treatment with AR-A014418, Tideglusib, VP2.51, and the peptide SCI, L803-mts, produced anti-depressive like results in the force swimming test (FST), which is a widely used test in evaluating anti-depressive activity of drugs (Gould et al., 2004; Kaidanovich-Beilin et al., 2004; Rosa et al., 2008; Perez-Domper et al., 2017). AF3581 was effective in two distinct models representing the down phase [using the chronic mild stress (CMS) model], or the up phase (using the resident intruder model). Accordingly, treatment with AF3581 improved depressive behavior and reduced aggressiveness (Capurro et al., 2020). In a serotonin deficit mouse model representing unipolar major depression, treatment with TDZD-8 alleviated depressive, anxious, and aggressive behaviors (Beaulieu et al., 2008). Notably, treatment with a different GSK-3 inhibitor, SB-216763, did not produce anti-depressive activity in the CMS model (Ma et al., 2013).

In summary, targeting GSK-3 may confer therapeutic advantages for the management of affective disorders including BD, as well as other depressive psychiatric conditions. Although GSK-3 is considered an *in vivo* target of lithium, it is clear that inhibition of GSK-3 may not completely recapitulate lithium activity as lithium has additional targets besides GSK-3.

### Schizophrenia

Schizophrenia is a neurodevelopmental mental illness that affects about 1% of the world’s population. The condition appears to be a multifactorial disorder with a strong genetic predisposition. In its most common form, schizophrenia presents with paranoid delusions and auditory hallucinations late in adolescence or in early adulthood (Insel, 2010). Early studies suggested that lithium might display efficacy for certain subgroups of schizophrenic patients (Hirschowitz et al., 1980, 1982; Zemlan et al., 1984; Shalev et al., 1987; Small et al., 2003). However, an updated meta-analysis (2015) of clinical trials in schizophrenia patients concluded that while lithium alone is not a sufficient therapy, it may be effective as an add-on treatment together with antipsychotics (Leucht et al., 2015). In a large survey of the Taiwanese population prescribed with lithium, it appeared that lithium was useful for treating schizoaffective disorder and certain subtypes of schizophrenia either as a monotherapy or adjunctive therapy (Luo et al., 2020). Indeed, co-treatment of lithium with antipsychotics such as clozapine, or divalproex, improved clinical symptoms and BPRS (brief psychiatric rating scale) scores in schizophrenic patients (Moldavsky et al., 1998; Bender et al., 2004; Kelly et al., 2006). Nevertheless, addition of a short term treatment with lithium (4 weeks) did not improve the condition of patients treated with antipsychotic medications (Collins et al., 1991). Finally, the results of a nationwide study in the Danish population, indicated that lithium therapy reduced the risk of suicide risk (Kessing et al., 2005), which is a key characteristic of schizophrenia (Fleischhacker et al., 2014).

Studies in animal models provided further evidence of the potential therapeutic benefits of lithium in treating schizophrenia-like conditions. In Akt1^(±)^ mice that mimic the genetic deficiency in schizophrenia patients (Tan et al., 2012), sub-chronic treatment with lithium alleviated psychomotor agitation and depressive behavior, and restored impaired sensorimotor gating (Luo et al., 2020). In a model of schizophrenia presenting with suicide-trait-related behaviors displayed by socially isolated mice, treatment with lithium reduced aggressiveness, impulsivity, and anxiety-like behavior (Deslauriers et al., 2016). However, despite these results, whether or not GSK-3 is a pathogenic target of schizophrenia is still in question. Some findings may link GSK-3 with the disease: first, GSK-3 is associated with schizophrenia susceptibility genes such as Akt1, DISC1, and TRAX (Translin-associated protein X) (Emamian et al., 2004; Freyberg et al., 2010; Lipina et al., 2011; Tan et al., 2012; Weng et al., 2018). In this context, an impaired Akt1-GSK-3 pathway (leading to activation of GSK-3) was found in the brain and peripheral lymphocytes of schizophrenia patients (Emamian et al., 2004), while DISC1 was originally discovered at the breakpoint of a balanced translocation t(1;11) (q42;q14.3) in a Scottish family with a high incidence of schizophrenia (Millar et al., 2000). Interaction of DISC1 with GSK-3α/β was shown to inhibit GSK-3 activity, and mutations in DISC1 can prevent GSK-3 inhibition (Mao et al., 2009; Lipina et al., 2011). In addition, the interaction of the DISC1/GSK-3 complex with TRAX, impairs the ability to repair damaged DNA (Chien et al., 2018; Weng et al., 2018); Second, a polymorphism of the GSK-3β gene was shown to be associated with schizophrenia (Tang et al., 2013; Yan et al., 2016); Third, the GSK-3 regulation of circadian genes already described (Lamont et al., 2010) may be related to the sleep disturbances seen in schizophrenic patients (Taliercio et al., 2020). Some case studies have described an elevation of GSK-3 activity in schizophrenia. For example, increased levels of GSK-3α/GSK-3β were detected in cerebrospinal fluid (CSF), and in platelets prepared from first-episode schizophrenia patients (Joaquim et al., 2018; Mohammadi et al., 2018). In addition, alterations in the PI3kinase/GSK-3 pathway were detected by RNA-sequence profiling in hiPSC-derived cells from schizophrenia patients (Stertz et al., 2021). Conversely, other reports detected no changes in GSK-3β mRNA levels in blood cells obtained from first-episode schizophrenia patients, and the levels of the protein were actually low in the frontal cortex and CSF of schizophrenia subjects (Kozlovsky et al., 2001; Zhang et al., 2021).

The use of GSK-3 inhibitors in animal models has demonstrated their ability to reverse disease symptoms. In DISC1 loss of function mice displaying schizophrenia-like behavior, disruption of GSK-3-DISC1 interactions, or treatment with SB-216763, reduced hyper-locomotion and produced anti-depressive like activity in FST (Mao et al., 2009). In mice expressing the DISC1*^L100P^* mutant, genetic inactivation of GSK-3α, or treatment with TDZD-8, lowered the observed hyperactivity and reversed schizophrenic like behavior (Lipina et al., 2011). In mice carrying a microdeletion on chromosome 22q11.2, a mutation that accounts for about 1%–2% of sporadic schizophrenia (Tamura et al., 2016), treatment with SB-216763 at the postnatal stage, rescued deficits in neural synchrony, task-related neural activity, and spatial working memory (Tamura et al., 2016). Similarly, treatment with BRD3731, a selective GSK-3β inhibitor, normalized abnormal cortical gamma oscillations and reversed deficits in working memory and acoustic startle of prepulse inhibition (PPI) in mice carrying a hypofunctional NMDS (N-methyl-d-aspartate) receptor (Nakao et al., 2020). In confirmation, subsequent knockdown of GSK-3β in these mice also reversed these behavioral deficits (Nakao et al., 2020).

Collectively, our basic understanding of the pathophysiology of schizophrenia is still lacking as well as the identification of suitable and optimal treatment. The clinical data suggest that GSK-3 may be a risk gene that contributes to susceptibility to schizophrenia, and studies in animal models provide some evidence that GSK-3 inhibition may improve schizophrenic symptoms including disease associated mood, sociability, aggressiveness, and cognitive dysfunctions.

## Cognitive Dysfunction

### Autism Spectrum Disorder

Autism Spectrum Disorder (ASD) is a multifactorial disorder that manifests in early childhood and is characterized by impaired social communication, repetitive behaviors, and restricted interests (Elsabbagh et al., 2012). A variety of potentially contributing genetic and/or environmental factors have been investigated in animal models of syndromic and non-syndromic ASD (Abrahams and Geschwind, 2008; Varghese et al., 2017). It has not proved possible to fully determine either the etiology of the condition, or an effective treatment for ASD. An estimated 10% of ASD cases are caused by an underlying genetic disorder, such as Fragile X Syndrome (FXS), Rett Syndrome (RTT), and Tuberous sclerosis (TSC) among others (Abrahams and Geschwind, 2008; Persico and Napolioni, 2013). Lithium has been suggested as a way to manage the mood disorders and behavioral symptoms typical of ASD, including euphoria, mania, or paranoia. In retrospective studies of children and adolescent patients with ASD, lithium improved mood disorders and maladaptive behavior (Siegel et al., 2014; Mintz and Hollenberg, 2019). In new born rats subjected to social isolation, chronic treatment with lithium overcame the autistic-like behavior, by a mechanism attributed to repair of neurogenesis and balancing of excitatory/inhibitory synaptic transmission (Wu et al., 2014). A phase III clinical study is currently planning to recruit ASD patients to evaluate the effect of 12 months of lithium treatment (NCT04623398).

Glycogen synthase kinase-3 (GSK-3) may increase ASD susceptibility due to the ability to regulate high-risk genes/pathways such as Wnt signaling/β-catenin, and the mTOR pathway (Kelleher and Bear, 2008; Rizk et al., 2021). Accordingly, elevated levels of GSK-3β together with increased levels of phosphorylated β-catenin were found in the amygdala of valproic acid (VPA)-induced ASD mice (prenatal administration of VPA confers a high risk of ASD in human and animals) (Wu et al., 2017). In this context, it is noteworthy that GSK-3 regulates the TSC -mTOR axis (Avrahami et al., 2020), and that high activity of the mTOR pathway was detected in the T cells of ASD children (Onore et al., 2017). Conversely, inhibition of mTOR by rapamycin was shown to improve social interaction deficits in ASD mice (Kotajima-Murakami et al., 2019). Notably, subjects with TSC (upstream to mTOR) are often diagnosed with ASD (de Vries et al., 2007).

Finally, Phase II Canadian clinical trials by AMO Pharma, where 83 subjects were treated with Tideglusib, reported “encouraging results” (NCT02586935) (Anagnostou, 2018). No other clinical data are available at this point.

### Autism Spectrum Disorder-Related Syndromes

#### Fragile X Syndrome

Fragile X Syndrome (FXS) is a genetic condition that represents the most common form of inherited autism and cognitive disability. It is caused by loss of function of the *FMR1* gene and reduced production of the encoded fragile X mental retardation protein (FMRP), an RNA binding protein that negatively regulates translation in neurons (Hagerman, 2006; Bhakar et al., 2012). Individuals with FXS have a characteristic facial morphology, and exhibit behavioral phenotypes including hyperactivity, attention and learning deficits, intellectual disability, anxiety, and seizure disorders (Hagerman, 2006). Lithium is considered a promising treatment in FXS, although the clinical data are rather limited (Liu and Smith, 2014). One pilot study reported the results of a 2-month treatment of 15 FXS subjects ages 6–23 with lithium, which revealed a significant improvement in CGIS (clinical global improvement scale) as well as improvements in hyperactivity, learning capability, abnormal vocalizations, self-abuse, and anxiety (Berry-Kravis et al., 2008). Studies in animal models have provided additional evidence for the therapeutic potential of lithium in treating FXS. The most popular model used is the FMR1 KO (Fmr^–/–^) mice, which exhibit similar symptoms to those seen in human FXS (van den Ouweland et al., 1994; Spencer et al., 2005). Indeed, treatment with lithium corrected locomotor hyperactivity and deficits in social interactions, and reduced anxiety. In addition, lithium treatment improved learning and memory, reduced the incidence of audiogenic seizures, and slowed the rates of protein synthesis that are typically exaggerated in FXS (Mines et al., 2010; Yuskaitis et al., 2010; Liu et al., 2011, 2012). We did not find reported evidence for alterations in GSK-3 in human FXS, although, studies in the Fmr^–/–^ mouse model suggested a role for GSK-3 in FXS pathogenesis. For example, GSK-3 activity was upregulated in the Fmr^–/–^ mouse brain (Min et al., 2009). Increased rates of protein synthesis (Sharma et al., 2010) could well be related to GSK-3 activation of mTOR (Avrahami et al., 2020). Constitutive GSK-3 activity in GSK-3 knock-in mice impaired social preferences, although the sociability behavior was normal (Mines et al., 2010). Another interesting finding is that phosphorylation of FXR1 (fragile X mental retardation syndrome-related protein) by GSK-3 has been suggested to contribute to disturbed mood and emotional instability (Del’Guidice et al., 2015). Moreover, functional genetic polymorphisms of FXR1 or GSK3β affect emotional stability (Khlghatyan et al., 2018).

Treatment with GSK-3 inhibitors including SB-216763, SB-415286, AR-A014418, CT99021, TDZD-8, VP0.7, and L803mts reversed the typical behavior phenotypes seen in the Fmr1^–/–^ mice. As a result, the animals’ performance in an assortment of memory and learning tasks improved, deficits in LTP (long term potentiation) were normalized, and the incidence of audiogenic seizures was reduced (Min et al., 2009; Guo et al., 2012; Chen et al., 2014; Franklin et al., 2014). A recent study that examined the impact of selective GSK3- isozyme inhibitors in Fmr1^–/–^ mice, reported that inhibition of GSK-3α by BRD0705, but not GSK-3β, corrected FXS symptoms in the Fmr1^–/–^ mice (McCamphill et al., 2020). However, the results of a different study, indicated that blockade of GSK-3β by siRNA diminished cognitive impairments in the Fmr1^–/–^ mice (Pardo et al., 2017). It is possible that pharmacological inhibition of GSK-3 does not produce equivalent results to those achieved by deletion of the protein expression, or alternatively that inhibition of GSK-3α may trigger inhibition of GSK-3β.

Altogether, although clinical data is still lacking at this point, results of studies in the FXS animal model provide strong evidence for the potential of GSK-3 inhibitors in improving cognitive and behavior deficits in FXS.

#### Rett Syndrome

Rett Syndrome (RTT) is an X chromosome-linked progressive neurodevelopmental disorder caused by loss-of-function mutations in the *MECP2* (methyl CpG-binding protein 2) gene (Amir et al., 1999; Cheadle et al., 2000; Ip et al., 2018). Rett Syndrome patients suffer from deficits of mental function, social withdrawal, and loss of previously acquired skills (e.g., loss of acquired motor skills and speech) (Hagberg, 2002). Abnormal dendrite morphology along with a decline in neuronal numbers are typical characteristics of RTT brains (Hagberg, 2002). A case study of a 20 month old RTT girl, reported improvements in the symptoms of irritability and hyperactivity, after treatment with lithium, but not with other antipsychotic medications used, such as zuclopenthixol and haloperidol (Kinay et al., 2016). It was suggested that upregulation of BDNF could be responsible for the therapeutic benefits observed (Tsai, 2006) and that this could be related to the neuroprotective effects of lithium and GSK-3 inhibition. In KO *MECP2* ^–/–^ mice that exhibit RTT phenotypes, GSK-3β activity was specifically elevated in the cerebellum (Jorge-Torres et al., 2018), which could be related to the aberrant mTOR regulation observed in a number of RTT models (Ricciardi et al., 2011). Importantly, treatment of *MECP2* ^–/–^ mice with SB-216763 alleviated disease symptoms in that it improved motor deficits, corrected abnormalities in the neuronal dendritic network, and reversed pro-inflammatory effects (Jorge-Torres et al., 2018). The anti-inflammatory effects were demonstrated by an increased in the level of expression of the cytokine IL-10, and reduced microglial infiltration into the cerebellum (Jorge-Torres et al., 2018). A different study found that BIO increased the expression levels of K^+^/Cl^–^ cotransporter 2 (KCC2) (Tang et al., 2019), a specific neuron ion transporter that is typically reduced in RTT (Dani et al., 2005). Subsequently, siRNA blockade of GSK-3β restored the normal expression levels of KCC2 and rescued morphological abnormalities observed in the MECP2 ^–/–^ neurons (Tang et al., 2019). Treatment with BIO also corrected impaired- GABAergic inhibition and- excitatory synaptic transmission in these neurons (Tang et al., 2019). The recovery in KCC2 expression levels by GSK-3 could be mediated by sirtuin 1 (Sirt1) (Tang et al., 2019), an important neuroprotective agent that was recently implicated as a GSK-3 downstream target (Rippin et al., 2021). Taken together, it is suggested that GSK-3/GSK-3-mediated pathways may be involved in the phenotype arising from *MECP2* deficiency, and that GSK-3 inhibition may be a potential therapy for treating the clinical symptoms of RTT.

#### Cyclin-Dependent Kinase Like-5 Deficiency Disorder

Cyclin-dependent kinase like-5 (CDKL5) disorder is a rare neurodevelopmental disease caused by mutations in the X-linked *CDKL5* gene. The condition is characterized by severe intellectual disability, early-onset epilepsy, motor rigidity, and RTT-like features (Tao et al., 2004). *CDKL5* knockout (CdkL5 ^–/–^) mice exhibit a *CDKL5* like phenotype and are a useful animal model to study this disorder (Amendola et al., 2014). It was suggested that the Akt/GSK-3 pathway may be responsible for the neuron loss and impaired dendritic development typically observed in the CDKL5 ^–/–^ hippocampus (Fuchs et al., 2014). In accordance with this notion, elevated levels of GSK-3β were found in the hippocampus of young and adult CDKL5 ^–/–^, together with increased phosphorylation of CRMP2 and lower levels of β-catenin (both GSK-3 targets) (Fuchs et al., 2014, 2018). Treatment with lithium restored survival and maturation of neural precursor cells (NPC) in the CDKL5 ^–/–^ brain (Fuchs et al., 2014). The GSK-3 inhibitors also produced a beneficial outcome in young CDKL5 ^–/–^ mice. Treatment with SB-216763 corrected hippocampal developmental defects, restored NPC survival and- maturation, and improved hippocampus dependent learning and memory (Fuchs et al., 2015). Studies with Tideglusib demonstrated that the drug was effective only when given during the juvenile period (Fuchs et al., 2018). This has led to the submission of a patent for Tideglusib as a treatment for CDKL5 disorders (WO/2017/153834). A dual inhibitor against GSK-3 and histone deacetylase 6 produced a significant improvement in neuron development and in cognitive function (Loi et al., 2021).

Thus, there may be a causal link between dysfunction of CDKL5 and increased GSK-3 activity that contributes to the neurodevelopmental defects expressed in the disease. However, treatment with a GSK-3 inhibitor may be effective only early in postnatal development.

#### Phelan-McDermid Syndrome

*SHANK3* is a leading candidate for an autism gene, with mutations occurring in between 1 and 2% of individuals with ASD (Han et al., 2013; Leblond et al., 2014). SHANK3 is a post-synaptic protein that is essential for proper synapse function, homeostatic plasticity, and structural organization of dendritic spines (Bey et al., 2018; Tatavarty et al., 2020). Phelan-McDermid Syndrome syndrome results from deletions in chromosome 22q13, or mutations in the *SHANK3* gene (Phelan and McDermid, 2012). Typical phenotypes of PMD may comprise symptoms characteristic of bipolar disorder and the autism spectrum, and often manifest in neonatal hypotonia, intellectual disability, absent or delayed speech, and a heightened risk of developing seizures (Phelan and McDermid, 2012). A case study of two patients with mutations or microdeletions in *SHANK3*, reported that treatment with lithium ameliorated the clinical deterioration, when compared to other psychotropic medications (Serret et al., 2015). The lithium treatment stabilized behavioral and mood symptoms and could even reverse the regression and catatonia seen in these patients (Serret et al., 2015). In another study with one PMD patient with a *SHANK3* mutation, co-treatment with lithium and olanzapine, stabilized mood behavior (Egger et al., 2017). A phase III clinical study currently plans to recruit ASD patients with PMD syndrome and to evaluate the effect of 12 months treatment with lithium (NCT04623398).

In a mouse model of *SHANK3* knockout mice (*SHANK3*^–/–^), lithium corrected the observed repetitive self-grooming phenotype, and restored intrinsic homeostatic plasticity and synaptic scaling in neurons isolated from the brain of these mice (Tatavarty et al., 2020). Interestingly, lithium did not rescue manic-like behavior or seizures in the *SHANK3*^–/–^ transgenic mice (Han et al., 2013). Inhibition of GSK-3 by BRD0320 rescued synaptic scaling in SHANK3^–/–^ neurons (Tatavarty et al., 2020), and inhibition with Tideglusib reduced anxiety (Furfaro, 2018), although this effect was seen only in this mouse model of ASD (out of four models tested).

#### Intellectual Disability

Intellectual Disability (ID) is characterized by significant limitations in both intellectual ability and adaptive behavior. The prevalence of the condition is estimated to be 1–3% but is extremely heterogeneous (van Bakel et al., 2014). Lithium is commonly used to manage behavioral problems in ID subjects (Deb et al., 2008). One study reported that treatment of ID children with a low-dose of lithium for 3 months improved the IQ and adaptive capacity scores without causing severe side effects (Yuan et al., 2018). The observation that Brazilian family members with severe ID associated with disruptive behavior, harbor a loss-of-function mutation in inositol monophosphatase 1 (IMPA1), a known target of lithium (Hallcher and Sherman, 1980; Figueiredo et al., 2016), supports the notion of lithium as a potential treatment for ID. Studies with GSK-3 inhibitors were not reported.

#### Conduct Disorder

Conduct Disorder (CD) is characterized by persistent aggressive and antisocial behavior that begins during childhood or adolescence and may continue into adulthood. It was initially reported that lithium reduced aggressiveness in hospitalized CD children (Campbell et al., 1984). This positive effect of lithium was further confirmed by subsequent reports that lithium treatment produced improvements in the hyperactivity, aggression, speech problems, unresponsiveness, and hostility in the majority (68–80%) of CD children treated (Campbell et al., 1995; Malone et al., 2000). A similar reduction in aggressive behavior was obtained in a retrospective study of CD children and adolescents treated with lithium either as a monotherapy, or in combination with typical antipsychotics (Masi et al., 2009). Studies with GSK-3 inhibitors were not reported.

#### Spinocerebellar Ataxias

Spinocerebellar Ataxias (SCAs) are heterogeneous autosomal dominant progressive neurodegenerative disorders caused by the expansion of polyglutamine in the ataxin protein product of the *ATXN* gene (Gatchel and Zoghbi, 2005). Although 47 types of spinocerebellar ataxia have been identified, types 1, 2, 3, 6, and 7 are the most prevalent, with type 3 being the most common (Burk et al., 2003). The diseases are typically characterized by a progressive loss of motor coordination, and the development of slurred speech, cognitive deficits, and brain atrophy. Two Phase II clinical trials in which patients with SCA2 and SCA3 (also known as Machado-Joseph disease) patients were treated with lithium for 48 weeks, concluded that lithium was well tolerated. In SCA2 patients there were no changes observed in brain volume or in the Rating and Assessment of Ataxia (SARA) scale, but there was some improvement in the Beck’s depression inventory (BDI-II), a validated scale for rating major depressive disorder (Sacca et al., 2015). Although lithium treatment did not reach the primary clinical endpoint in a study with SCA3 patients, there was a significant slowing of disease progression (Saute et al., 2014). Both reports recommended further continuation of studies with lithium (Sacca et al., 2015; Saute et al., 2015).

Similarly, treatment with lithium improved motor coordination, learning, and memory, and attenuated reduced dendritic branching in a transgenic SCA1 mouse model (Watase et al., 2007). In contrast, lithium did not produce overall beneficial effects in motor performance in a SCA3 mice model, although there was some reduction of tremors (Duarte-Silva et al., 2014). In a Drosophila model of SCA3, chronic treatment with lithium prevented eye depigmentation, alleviated locomotor disability, and extended the median life span (Jia et al., 2013). Interestingly, lithium in combination with mTOR inhibition significantly decreased the level of expression of mutant atexin-3 and reduced the amount of nuclear aggregates, although it did not rescue motor impairments and did cause some neurotoxicity (Duarte-Silva et al., 2014). Studies with GSK-3 inhibitors were not reported.

#### Down Syndrome

Down Syndrome (DS) is the most common genetic disorder associated with cognitive disability and is caused by trisomy of chromosome 21 (HSA21). The typical DS phenotype features impairments in motor function, a decrease in the number of neurons and brain volume, and a high prevalence of dementia in patients above 35 years of age (Mann, 1988; Antonarakis et al., 2020). The few clinical studies of treating DS with lithium therapy in the literature, have provided only circumstantial evidence. One study reported that lithium enhanced proliferation and restored the responsiveness to PHA (Phytohemagglutinin) in lymphocytes of aging DS subjects (Licastro et al., 1983). This could well be relevant to the reduced life span, which is a typical feature of DS. Another study, described lower plasma concentrations of metal ions including lithium, zinc, and copper in DS samples (Bruhl et al., 1987). This could hint at possible upregulation of GSK-3 in the DS brain, as these cations may act *in vivo* as GSK-3 inhibitors (Ilouz et al., 2002; O’Brien and Klein, 2009). Indirect evidence for the potential of treatment of lithium may be provided by the findings that increased levels of myo-inositol in the DS brain correlate with reduced cognitive function (Huang et al., 1999). Treatment with lithium could thus correct this defect by managing the brain inositol pool through inhibition of IMPase (Manji et al., 1995). Finally, DS patients who experience episodes of mania may be treated with lithium (Cooper and Collacott, 1993). Little is known about GSK-3 in human DS. One study reported increased GSK-3β activity in trisomic fetal human brains (Trazzi et al., 2014). Another indirect connection is the missense mutations of GSK-3 phosphorylation sites found in *MAF* (v-maf avian musculoaponeurotic fibrosarcoma oncogene homolog), the causative gene of Ayme-Gripp syndrome, where the facial features are similar to those seen in DS (Niceta et al., 2015).

From a mechanistic point of view, the ability of lithium, or GSK-3 inhibition to promote neurogenesis (Hur and Zhou, 2010; Haupt et al., 2021), may support the suggestion that decreased neurogenesis is a dominant factor in the brain hypotrophy and cognitive decline seen in DS (Antonarakis et al., 2020). One clinical study reported that administration of micro-doses of lithium reduced the progression of AD in DS patients (Priebe and Kanzawa, 2020). In agreement, chronic lithium treatment restored neurogenesis in the hippocampus vertical zone in the segmental trisomy mouse model, Ts65Dn, and improved hippocampal-dependent cognitive functions (Bianchi et al., 2010; Contestabile et al., 2013). Lithium treatment also restored the number of NPC by stimulating the Wnt/β catenin pathway (Guidi et al., 2017), and increased the activity of GSK-3β in the cells by indirect interaction with the APP intracellular fragment (AICD), a molecule that is typically increased in DS (due to the triplicated APP gene) (Trazzi et al., 2014). Activation of GSK-3β impaired NPC proliferation, cell fate specification, and cell maturation (Trazzi et al., 2014).

In agreement with the human findings (Huang et al., 1999), higher than normal levels of myo-inositol were present in the Ts65Dn brain, and were reduced by lithium (Huang et al., 2000). Subsequently, increased GSK-3β activity (manifested by increased tyrosine phosphorylation) was detected in brains of the Ts1Cje mouse line that carries a trisomic segment lacking *APP* and *SOD1* (Shukkur et al., 2006). This was accompanied by hyperphosphorylation of tau, a known GSK-3 target, as well as mitochondrial dysfunction and oxidative stress (Shukkur et al., 2006). Studies with Tideglusib, however, did not detect improvement in memory or reduced neurogenesis in the Ts65Dn mice (Giacomini, 2018).

In summary, increased GSK-3 activity is found in the human fetus and in adult DS models. It may thus be responsible, at least in part, for the impaired brain development and reduced neurogenesis seen in DS. It will be of great interest to test GSK-3 inhibitors in DS models.

## Neurodegenerative Diseases

### Alzheimer’s Disease and Other Tauopathies

Alzheimer’s disease (AD) is the most prevalent neurodegenerative disorder in the world, and is a leading cause of dementia in late adult life. Alzheimer’s disease pathogenesis is characterized by accumulation of extracellular aggregates of amyloid β (Aβ) plaques, and neurofibrillary tangles composed of hyperphosphorylated tau proteins. These eventually lead to the synaptic and neuronal loss, that are responsible for the slow and gradual deterioration of memory and the decline in language, personality, and cognitive control (Holtzman et al., 2012; Long and Holtzman, 2019). Cholinesterase inhibitors are the main treatment used to delay symptoms, and recently immunotherapy in the form of a monoclonal antibody (aducanumab) directed toward beta amyloid plaques, has been approved for treating AD (Mullard, 2021).

Clinical trials examining the potential benefits of lithium on cognitive abilities in AD and dementia patients, reported mixed results. A meta-analysis of three studies of randomized placebo-controlled clinical trials of AD and mild cognitive impairments (MCI), with a total of 232 subjects, showed that lithium decreased the progress of cognitive decline, with better results in the AD subgroup (Matsunaga et al., 2015). Study of prescribing lithium over a period of 10 years for patients with diagnosed dementia in Danish population, concluded that continued lithium treatment reduced the rate of dementia or AD incidence (Kessing et al., 2008). Similarly, chronic treatment of elderly bipolar patients with lithium lowered the incidence of AD compared to individuals not or minimally exposed to lithium during their lifetime (Nunes et al., 2007). Similarly, treatment of amnestic MCI patients with lithium for a year resulted in a better performance on memory and attention tests as well as a reduction in phosphorylated tau in the CSF compared to controls (Forlenza et al., 2016). Finally, low dose treatment of lithium was effective in preventing agitation and violent behavior in AD or frontotemporal dementia patients (Devanand et al., 2017). It is noteworthy that micro-doses of lithium delayed the progression of AD in DS patients (Priebe and Kanzawa, 2020). The efficacy of micro-dose lithium treatment in preventing cognitive loss in AD patients suggests that low concentrations of lithium may be a favorable approach (Nunes et al., 2013). Negative results, however, were obtained with short-term lithium treatment (10 weeks) in mild AD (Hampel et al., 2009). Another study was discontinued after 16–36 weeks of treatment due to the vulnerability of the elderly patients to lithium side effects (Macdonald et al., 2008). Studies of lithium treatment in animal models of AD produced important therapeutic data that has been well summarized elsewhere (Morris and Berk, 2016; Kerr et al., 2018; Damri et al., 2020). Taken together, the clinical results suggest that long-term lithium treatment is required for a beneficial effect, and that it may be particularly effective when started in the early stages of disease. Importantly, the use of microdose lithium may overcome the toxicity problems that have forced the discontinuation of certain previous trials (Morris and Berk, 2016; Damri et al., 2020).

The contribution of GSK-3 to AD pathogenesis is well documented and has been proven to work through multiple mechanisms. These include regulation of amyloid precursor (APP) processing, generation of β-amyloid, tau hyper-phosphorylation to form neurofibrillary tangles (NFT), regulation of axonal transport, autophagy, and inhibition of the Wnt signaling pathway (Phiel et al., 2003; Ly et al., 2013; Sanchez-Cruz et al., 2019; Rippin and Eldar-Finkelman, 2021; Sayas and Avila, 2021). Recent results have also implicated GSK-3 in ER stress linked to memory deficits (Lin et al., 2018), and impairment in neuronal oscillation linked to cognitive deficits (Nguyen et al., 2017). Studies reported increased GSK-3 expression levels in human AD brain extracts and demonstrated GSK-3 co-localization with NFT (Pei et al., 1997; Ferrer et al., 2002; Leroy et al., 2007). Increased GSK-3 activity was also detected in white blood cells and in platelets obtained from AD and MCI patients (Hye et al., 2005; Armentero et al., 2011; Forlenza et al., 2011). Interestingly, a reduction in GSK-3β levels has also been reported in such patients and was more pronounced in MCI patients than AD patients (Marksteiner and Humpel, 2009). In this context, genetic polymorphisms reveal an association between GSK-3β and tau genes that increases the risk for AD (Kwok et al., 2008).

Studies in animal models of AD provide strong evidence for the contribution of GSK-3 to AD pathogenesis. In a genetic-based approach, overexpression of GSK-3β resulted in increases in tau phosphorylation and gliosis, impaired LTP, and in a small size brain in transgenic mice (Spittaels et al., 2000; Lucas et al., 2001; Hernandez et al., 2002; Engel et al., 2006). Conditional expression of GSK-3β in the mouse brain decreased postsynaptic density number and reduced the volume in hippocampal granule neurons, a phenomenon associated with cognitive impairment (Llorens-Martin et al., 2013). The toxicity of the GSK-3-tau axis and its relevance to AD pathogenesis was confirmed in tau knockout mice (Gomez de Barreda et al., 2011). Conversely, genetic deletion of GSK-3 isozymes reduced β-amyloid load and reduced neurofibrillary tangles in the AD mouse brain (Hurtado et al., 2012; Ly et al., 2013). Similarly, blockade of GSK-3β by antisense oligonucleotides ameliorated AD symptoms in SAMP8 mice, a mouse model that displays increased Aβ levels, tau hyperphosphorylation, and cognitive deficits (Farr et al., 2014). Finally, activation of GSK-3β by β-amyloid, or, by PI3 kinase inhibitors, aggravated neuronal tauopathy, and disrupted axonal transport and cholinergic homoeostasis, respectively (Terwel et al., 2008; Wang Y. et al., 2017).

The promising results in AD models make GSK-3 a favorite kinase target in treating AD (Eldar-Finkelman and Martinez, 2011; King et al., 2014; Tolosa et al., 2014; Lovestone et al., 2015; Palomo and Martinez, 2017; Roca and Campillo, 2020). Here we present a summary of studies with GSK-3 inhibitors, starting with those that reached the clinic. The clinically approved drug Tideglusib, was initially tested in a number of animals models. Oral administration of the drug to mice co-expressing mutant APP with triple mutated human tau (APP*^SW^*/tau*^VLW^*) decreased Aβ deposition, reduced tau phosphorylation, and improved memory deficits (Sereno et al., 2009). In the rat hippocampus, Tideglusib prevented inflammation and edema formation (Luna-Medina et al., 2007), and increased adult hippocampal neurogenesis (Morales-Garcia et al., 2012). Studies in the clinic showed safety but no efficacy: in a small pilot study, Tideglusib showed good tolerability in mild-moderate AD patients (NCT00948259), with a significant improvement in the Mental Status Examination (MMSE) test (del Ser et al., 2013). In contrast, treatment with Tideglusib for 26 weeks did not prevent cognitive decline in the phase II ARGO study (NCT01350362) that included 306 mild-moderate AD patients (Lovestone et al., 2015). Similarly, the TAUROS phase II study that included 146 mild-to-moderate progressive supranuclear palsy (PSP) patients treated with Tideglusib for 52 weeks, did not reach the primary or secondary end points (Tolosa et al., 2014), although there was some reduction in brain atrophy observed (Hoglinger et al., 2014). The conclusions from these studies is that the drug is safe but that clinical efficacy may require a longer duration of treatment (Lovestone et al., 2015). Despite this promise, clinical studies of Tideglusib in AD have been discontinued, and the drug is currently being tested by AMO Pharma for alternative indications, including myotonic dystrophy and autism.

The thiazole compound AR-A014418 developed by AstraZeneca as a selective GSK-3 inhibitor was initially reported in 2003 (Bhat et al., 2003). Treatment with AR-A014418 reduced tau phosphorylation in cells and prevented β-amyloid-induced neurotoxicity in hippocampal slices (Bhat et al., 2003). However, PET scanning indicated that AR-A014418 is not brain penetrable (Vasdev et al., 2005) and the drug was abandoned due to poor physico-chemical and pharmacological properties (Bhat et al., 2018). The preclinical and clinical development process of AstraZeneca drug candidates over the last two decades has been well summarized in the literature (Bhat et al., 2018). The bottom line is that despite the excellent pharmacological properties of the designated inhibitors, the associated severe side effects do not support further clinical development. The leading candidates, namely AZD2858 from the pyrazine class, and AZD1080 belonging to the oxindole pyridine series, are both orally bioactive with excellent drug properties (Bhat et al., 2018). However, while AZD2858 exhibited dose-dependent inhibition of tau hyper-phosphorylation and reduced gliosis in the rodent hippocampus, it also caused a rapid and robust increase in bone formation in pre-clinical toxicology studies (Bhat et al., 2018). AZD1080, which was extremely efficacious at inhibiting tau phosphorylation, and could reverse deficits in synaptic plasticity (Georgievska et al., 2013), was well tolerated in Phase I clinical studies in healthy volunteers. Unfortunately, chronic dosing induced severe histopathological changes in the gall bladder in dogs, which forced the discontinuation of Phase II clinical trials (Bhat et al., 2018).

Although other GSK-3 inhibitors did not enter clinical trials for AD, it is instructive to describe their impact in animal models. SB-216763 and SB-415286 were initially noted for their anti-apoptotic activity in cells (Coghlan et al., 2000), and SB-216763 produced some therapeutic activity that correlated with attenuation of AD symptoms. Thus, in mice infused with β-amyloid, SB-216763 protected against neuronal damage and prevented aberrant dendritic morphology (Hu et al., 2009). The results of subsequent studies indicated that administration of SB-216763 to rats, improved spatial learning memory and induced theta frequency oscillations, known to be important for cognition (Nguyen et al., 2017). The drug was also shown to reduced tau phosphorylation in the rodent brain (Selenica et al., 2007; Xiong et al., 2013). Intracerebroventricular (icv) infusion of SB-216763 in the rat, prevented angiotensin II-induced tau phosphorylation and the resultant impact on cognitive impairments (Tian et al., 2012). In addition, SB-216763 exhibited good brain uptake in rodents and rhesus macaques. The observation that the drug is not selective and also functioned against other structurally similar kinases (Wang et al., 2011; Li et al., 2015) may at least partially explain why the clinical program was not advanced further.

Another material, developed by Sanofi, namely SAR502250, prevented neuron degeneration in transgenic mice expressing human tau*^P301L^* mutant and improved the cognitive deficits in aged APP*^SW^*/Tau*^VLW^* mice (Griebel et al., 2019). However, no other reports were found in the literature concerning this compound.

A highly selective GSK-3 inhibitor, PF-04802367 developed by Pfizer, was first identified as a high-throughput screening hit with good ADME profile. PET scans revealed that the drug was able to enter the brain of rats and rhesus macaques, and could reduce tau phosphorylation *in vivo* (Liang et al., 2016). The radiolabeled molecule could be used as a radiotracer for brain-GSK-3 (Liang et al., 2016), and represents a member of a family of molecules being developed as GSK-3 radiotracers for human imaging (Varlow et al., 2021).

Isonicotinamides are a class of very specific GSK-3 inhibitors developed by Bristol Myers Co., with a unique mode of binding to the ATP binding site. They have the advantage that they are orally bioavailable and proved able to reduce tau hyper-phosphorylation in triple-transgenic mice (3 × Tg-AD) harboring mutations in presenilin1 (PS1), APP, and tau (PS1*^M146V^*, APP*^Swe^*, tau*^P301L^*) (Luo et al., 2016).

The triazolo[4,3-*a*]pyridin-3(2*H*)-one derivatives termed C-7a and C7-b, were developed through docking studies with staurosporine and SAR analysis, and serve as orally effective GSK-3 inhibitors (Noh et al., 2013). The drugs decreased β-amyloid loads and tau phosphorylation, and improved short term memory in the 3xTg-AD mice (Noh et al., 2013). Another orally bioavailable MMBO molecule (2-methyl-5-(3-{4-[(S)-methylsulfinyl]phenyl}-1-benzofuran-5-yl)-1,3,4-oxadiazole) produced similar results in that it suppressed tau pathology and improved cognitive and memory deficits in 3xTg-AD mice (Onishi et al., 2011). Finally, the use of isoorientin, analogs were implicated in AD therapy (Song et al., 2017).

In consideration of the complicated interrelations between multiple pathways involved, it may be argued that a multitarget strategy should be a more effective method to manage AD disease (Zhang et al., 2019). As part of this approach, the dual inhibition of acetylcholinesterase (AChE) and GSK-3 was predicted to produce beneficial effects by preventing acetylcholine decline and the toxic effect of hyperactive GSK-3 (Zhang et al., 2019). Strategic design was employed to incorporate two distinct pharmacophores thought to be important for the inhibition of AChE and GSK-3, and the result was tacrine-pyrimidone hybrid compounds (Yao et al., 2021). The chosen compound, 27g, entered the brain *in vivo*, and provided neuroprotection against glyceraldehyde-induced neurite damage in cells, and scopolamine-induced cognition-impairment (Yao et al., 2021).

The use of the peptide SCIs L803-mts and L807-mts reduced the β-amyloid loads, improved memory and sociability, and reduced inflammation in 5XFAD mice that carry mutations in PS1 and APP (Avrahami et al., 2013; Licht-Murava et al., 2016).

In summary, the results indicate that GSK-3 inhibitors have great therapeutic potential in AD. This is well reflected in the pie-chart (Figure 1), showing that the majority of experiments with GSK-3 inhibitors were in AD models. The failure to demonstrate efficacy in AD patients is somewhat disappointing, although since Tideglusib is the only compound tested in humans to date, it is possible that other inhibitors with a different inhibition modality and good safety and pharmacokinetic properties and safety will prove more effective. It is also possible that the complex mechanisms of AD pathogenesis in humans will require simultaneous targeting of multiple molecules, including but not limited to GSK-3.

### Parkinson’s Disease

Parkinson’s disease (PD) is caused by loss of neuronal substantia nigra, leading to striatal dopamine deficiency. A key pathological feature of PD pathology is the build-up of synuclein aggregates in the form of Lewy bodies. Major clinical symptoms include resting tremor, rigidity, and bradykinesia (Kalia and Lang, 2015). Tau pathologies have also been implicated in PD (Lei et al., 2010). We found one ongoing trial using lithium in PD (NCT04273932) reported. The potential contribution of GSK-3 to PD pathogenesis has been well documented (Golpich et al., 2015). Accordingly, increased levels of expression of GSK-3β and tyrosine phosphorylation (reflecting GSK-3 activation) were found in postmortem PD brains (Duka et al., 2009; Nagao and Hayashi, 2009; Wills et al., 2010), and both inactive and active forms of GSK-3β were co-localized with α-synuclein (Nagao and Hayashi, 2009). Increased levels of GSK-3β protein were also reported in peripheral blood lymphocytes of PD patients (Armentero et al., 2011). Genetic variability of the GSK-3β gene is considered a risk factor for PD. Functional single nucleotide polymorphisms (SNPs) affecting gene transcription and splicing were identified in the GSK-3β gene of Australian and Chinese PD cohorts (Kwok et al., 2005). Similarly, a GSK-3β polymorphism was found to be overrepresented in Greek and Indian PD patients (Kalinderi et al., 2011; Das et al., 2012). Furthermore, synergistic interactions between the GSK-3β gene and tau, CDK-5, and oxidative stress related genes, significantly increased PD risk (Kwok et al., 2005; Garcia-Gorostiaga et al., 2009; Das et al., 2012). In animal models, constitutively active GSK-3β was shown to enhance the loss of dopaminergic neurons and to accelerate the accumulation of α-synuclein aggregates (Credle et al., 2015). Conversely, conditional depletion of GSK-3β, but not GSK-3α, rescued dopaminergic neurodegeneration in the MTPT (1-methyl-4-phenyl-1,2,3,6-tetrahydropyridine)- induced PD model (Li et al., 2020). It is noteworthy, that MTPT activates GSK-3β in dopaminergic neurons (Wang et al., 2007). Additional studies demonstrated that GSK-3 activity is controlled by factors/signaling pathways affecting PD. For example, the putamen dopamine D3 receptor was upregulated by the Akt/GSK-3β pathway in capuchin monkeys with tardive dyskinesia, a typical feature of PD (Hernandez et al., 2019). Inhibition of GSK-3 by the Dapagliflozin, or, insulin provided neuroprotection and ameliorated cognitive deficits in the rotenone-induced, or, 6-hydroxydopamine (6-OHDA)-induced PD models (Li et al., 2020; Arab et al., 2021). Interestingly, L-DOPA neurotoxicity as manifested by induced dyskinesia, was associated with GSK-3 activation, which raises some concerns about the use of this drug (Choi and Koh, 2018).

The available data suggest that inhibition of GSK-3 may protect dopaminergic neurons. Notably, studies with lithium that produced significant therapeutic benefits in PD animal models are summarized elsewhere (Haupt et al., 2021; Vallee et al., 2021). In the MTPT- induced PD model, treatment with indirubin-3′-oxime, AR-A014418, or high doses of Tideglusib, alleviated dopaminergic neuron loss and improved motor capability (Wang et al., 2007; Li et al., 2020), while treatment with SC001 prevented dopaminergic neurodegeneration and reduced microglia activation in two models of PD induced by either 6-OHDA or lipopolysaccharide (LPS) (Morales-Garcia et al., 2013). In accordance with the assumption of insufficient formation or accelerated degeneration of newly formed neurons in PD, SB216763 treatment of rats with PD induced by 6-OHDA rats, enhanced self-renewal, proliferation, and differentiation of hippocampal neural stem cells (NSC) (Singh et al., 2018). Treatment of transgenic mice that carry α-synuclein*^A53T^* with TWS119 improved the motor ability on the rotarod and open field tests (Ho et al., 2020). Finally, treating DAT-KO mice with a variety of GSK-3 inhibitors including SB-216763, Alsterpaullone, indirubin-3-monoxime, and TDZD-8, reduced the dopamine-dependent locomotor hyperactivity and stereotypic movements present due to the lack of dopamine receptors (Beaulieu et al., 2004). It is noteworthy that these mice exhibited elevated striatal GSK-3α/β activity (Beaulieu et al., 2004).

### Huntington’s Disease

Huntington’s Disease (HD) is an inherited disorder caused by a CAG trinucleotide repeat expansion (an expanded polyglutamine tract) in the huntingtin gene (*Htt*). The mutant Huntingtin protein (mHtt), and, in particular, the N-terminal fragments, tend to misfold and form aggregates with neurotoxic activity in the cytoplasm and nuclei (Bates et al., 2015; McColgan and Tabrizi, 2018). Huntington’s Disease is a member of the family of neurodegenerative triplet repeat disorders (Ashkenazi et al., 2017) and is characterized by progressive motor dysfunction, emotional disturbances, and dementia. Reducing mHtt aggregates is considered a promising therapy (Barker et al., 2020).

Early on in the 1970s, a number of clinical trials administered lithium for short time periods to small groups of HD patients. No improvement was observed in involuntary movements, hyperkinesia, motor skills, or in the ability to perform everyday tasks (Anden et al., 1973; Aminoff and Marshall, 1974; Leonard et al., 1975). Other trials did report improvements in mood disorders in lithium treated HD patients (Dalen, 1973; Mattsson, 1973) and newer clinical trials conducted in the last decade reported that lithium improved mood and behavior with no worsening of psychiatric symptoms or choreic movements (Danivas et al., 2013). Administration of lithium to suicidal HD patients treated with tetrabenazine (which increases the risk for depression or suicide) eliminated suicidal symptoms and improved depressive behavior (Raja et al., 2013). The potential of lithium in treating HD has also described (Scheuing et al., 2014).

From a mechanistic point of view, inhibition GSK-3 may reduce mHtt toxicity via the ability to increase the amount of heat shock proteins [activation of heat shock factor-1 (HSF-1)], by activating autophagy, and by reducing tau pathology (Carmichael et al., 2002; L’Episcopo et al., 2016; Menzies et al., 2017; Rippin et al., 2021). There is no evidence for increased GSK-3 activity in the HD brain. In fact, studies of postmortem HD brain revealed an overall decrease in the amount of GSK-3 reflecting the decreased activity (Lim et al., 2014; Fernandez-Nogales et al., 2015). It is possible that this reflects a reduction in the expression levels of all proteins at this stage of the disease, making it difficult to conclude whether these results mean that GSK-3 directly contributes to human HD.

A number of HD mouse models have been described (Farshim and Bates, 2018). In the R6/2 model where the 1*^st^* exon of mHtt is expressed, treatment with lithium improved the rotarod performance, but did not affect survival (Wood and Morton, 2003). Treatment of YAC128 mice that express human mHtt, with a new low-dose lithium formulation (NP03), ameliorated the deficits in striatal volume, improved motor function, and restored the expression of the DARPP-32 dopamine signaling protein, which is a specific marker for neuron loss (Pouladi et al., 2012). Similarly, combined therapy with lithium and VPA alleviated locomotor deficits in both YAC128 and N171-82Q mice (where the latter express the N-terminal fragment of human mHtt), and improved coordination and anxiety behavior (Chiu et al., 2011). In a quinolinic acid- HD-induced rat model (in which the excitotoxin is infused into the striatum), lithium reduced striatal lesions, and stimulated neuronal and astroglial progenitor proliferation at the injection site (Wei et al., 2001; Senatorov et al., 2004). Studies in flies have provided further evidence for the importance of the mTOR/autophagy pathway in protection against mHtt toxicity (Berger et al., 2006; Sarkar et al., 2008). Two studies were performed with GSK-3 inhibitors. The results of the first study indicated that SB216763 reduced mHtt cytotoxicity (Carmichael et al., 2002; Valencia et al., 2012). The second, demonstrated *in vivo* efficacy of the GSK-3 SCI, L807-mts, in R6/2 mice. Treatment with L807-mts reduced the amounts of striatal mHtt aggregates, improved motor and coordination ability, and ameliorated clasping episodes (Rippin et al., 2021). These therapeutic effects were attributed to activation of autophagy and increased expression of neuroprotective factors including Sirt1 and BDNF (Rippin et al., 2021).

In summary, accumulated evidence suggests that GSK-3 indeed plays a role in HD pathogenesis. The observed impact on degradative pathways such as autophagy and activation of neuroprotective factors such as HSF-1, BDNF, and Sirt1 among others, make GSK-3 inhibition a particularly attractive modality for treating HD, even though only one study has used a GSK-3 inhibitor in an *in vivo* HD model. Notably, studies in the human brain, may not support a toxic role of GSK-3 in human HD. It is possible that GSK-3 is effective only in the earlier stages of the disease but more studies will be required to clarify this issue.

### Amyotrophic Lateral Sclerosis

Amyotrophic Lateral Sclerosis (ALS) is a progressive and ultimately fatal adult-onset neurodegenerative disorder characterized pathologically by loss of spinal and cranial motoneurons, as well as corticospinal tract neurons (Shaw et al., 2001; Taylor et al., 2016). Heterogeneous genetic variations are found in both familial and sporadic ALS. In familial ALS (5–10% cases), causative mutations have been identified in superoxide dismutase (*SOD1*) and TDP-43 (TAR DNA binding protein) (Taylor et al., 2016). Administration of lithium was reported to delay disease progression in ALS patients, as measured by changes in muscle strength and preservation of pulmonary function (Fornai et al., 2008). Unfortunately, additional clinical trials did not report any benefits of lithium treatment (Aggarwal et al., 2010; Chio et al., 2010; Miller et al., 2011; Group et al., 2013). It is possible that the genetic heterogeneity of the disease plays a role in drug response. Indeed, meta-analysis of ALS patients with different genotypes showed different responses to lithium (van Eijk et al., 2017). In addition, some trials also raised safety concerns about lithium (Miller et al., 2011).

Human and animals studies suggest a possible role for GSK-3 in ALS (Choi et al., 2020), since GSK-3 is up-regulated in the brain and spinal cord of ALS patients (Hu et al., 2003; Yang et al., 2008). Results in animal models have demonstrated that GSK-3 phosphorylates TDP-43, and that deletion of the GSK3β provided protection against TDP-43-induced toxicity (Moujalled et al., 2013).

Studies with lithium showed beneficial results in ALS models, where lithium delayed the onset of paralysis and muscle weakness (Shin J. et al., 2007; Feng et al., 2008; Fornai et al., 2008). Addition of VPA further improved the results (Feng et al., 2008). Treatment of transgenic mice expressing the SOD1*^G93A^* mutant with AR-A014418, attenuated motor impairments, increased motor neuron survival, reduced inflammation, and delayed lethality (Koh et al., 2007; Ahn et al., 2012). Similarly, administration of an oral inhibitor JGK-263, provided neuroprotection, improved motor ability and increased survival in the SOD1*^G93A^* mice (Ahn et al., 2012). Treatment with VP2.51 resulted in complete recovery of neurological symptoms in a rat model of ALS induced by beta-N-methylamino-L-alanine (L-BMAA) (de Munck et al., 2016). Finally, dual inhibition of GSK-3 and CDK-5 protected against motor neuron degeneration in a zebrafish model (Reinhardt et al., 2019).

Taken together, there are multiple indications that inhibition of GSK-3 may prevent motor neuron degeneration and delay ALS disease progression. However, the real therapeutic potential in patients remains to be elucidated.

### Multiple Sclerosis

Multiple Sclerosis (MS) is a chronic autoimmune demyelinating disease of the CNS that leads to progressive and severe neurological disability. In MS, the myelin-forming oligodendrocytes (OL), which are the products of differentiation of oligodendrocyte progenitor cells (OPC) in the subventrical zone, are the targets of inflammatory and immune attacks (McFarland and Martin, 2007; Goldenberg, 2012; Bergles and Richardson, 2015). As a result, remyelination- is inefficient, due to impairments in OPC differentiation. Thus, promoting OPC maturation is considered a valuable therapeutic approach that may enhance neuroprotection and remyelination. A number of clinical trials have been conducted with lithium treatment. A retrospective study of US veterans with MS did not detect a consistent improvement in disease symptoms after lithium treatment (Rinker et al., 2013), although a later study reported that low-dose lithium treatment was well tolerated by MS patients and provided a “favorable” improvement on a quality of life scale (QOL) (Rinker et al., 2020). Lithium could also improve mania episodes in MS patients (Yang and Wichser, 2020). One of the few studies examining GSK-3 in human MS reported increased expression of GSK-3β in the corpus callosum and cerebral cortex of chronic progressive MS patients (Booth et al., 2005). Another study reported that a genetic polymorphism in the promoter region of the GSK-3β gene could be associated with susceptibility for MS (Galimberti et al., 2011).

The contribution of GSK-3 to MS pathogenesis may well be attributed to the pro-inflammatory activity and the ability to inhibit OPC differentiation. This was demonstrated in *in vivo* studies where GSK-3 induced the production of inflammatory cytokines via regulation of Toll-like receptors (Martin et al., 2005). Conversely, conditional depletion of GSK3β in mature OL, reduced de-myelination and activated glial cells in the acute cuprizone-induced de-myelination mouse model (Xing et al., 2018), while, CDK-5-induced OPC maturation was restored by GSK-3 inhibition (Luo et al., 2014). Finally, GSK-3 was shown to regulate the differentiation of pathogenic Th1 and Th17 T-helper cells (Beurel et al., 2013).

The most widely used animal model of MS is experimental autoimmune encephalomyelitis (EAE), in which the disease symptoms mimic MS pathology (Constantinescu et al., 2011). Studies in the EAE model confirmed the devastating role of GSK-3 in MS. Increased GSK-3β activity was measured in the EAE spinal cord (De Sarno et al., 2008; Ahn et al., 2017), and expression of constitutively active GSK3α/β enhanced EAE severity (De Sarno et al., 2008). Conversely, maintaining the inactive form of GSK-3β preserved the normal physiology of the spinal cord and enabled efficient stem cell therapy (Tafreshi et al., 2014).

Lithium therapy in MS animals was reported as early as in 1992, when injections of lithium were shown to inhibit the development of EAE in rats (Levine and Saltzman, 1992). Later studies supported the efficacy of lithium in MS in that lithium treatment delayed the onset of paralysis, reduced- demyelination and- leukocyte infiltration into the spinal cord, and blocked relapse in rodents with EAE (De Sarno et al., 2008; Ahn et al., 2017). Treatment of EAE with GSK-3 inhibitors including TDZD-8, VP2.51, VP2.7, and L803-mts produced similar results (Beurel et al., 2013). In a lysolecithin-induced demyelination model, treatment with ARA-014418, indirubin, or L803-mts induced OPC differentiation during brain development, and promoted regeneration of OL and re-myelination in the adult (Azim and Butt, 2011). Similarly, treatment with VP3.15, which is a dual inhibitor for phosphodiesterase (PDE) and GSK-3, enhanced OPC differentiation and restored remyelination in the cuprizone-induced MS model (Medina-Rodriguez et al., 2017). Interestingly, the results indicated that TDZD-8 alone did not affect OPC maturation, but did preserve cell survival (Medina-Rodriguez et al., 2017).

Taken together, inhibition of GSK-3 with the resulting anti-inflammatory and- pro-remyelination activity, may be a promising approach to treating MS. Further proof will require additional clinical trials to investigate lithium and FDA approved GSK-3 inhibitors.

## Additional Neurological-Related Conditions

Information about the use of GSK-3 inhibitors in additional neuron-related conditions can be found in the literature. Due to lack of space, we briefly summarize the studies conducted with GSK-3 inhibitors. Therapeutic benefits of GSK-3 inhibitors have been observed in the treatment of injuries to the brain and spinal cord, as well as in brain ischemia. Administration of the inhibitors to animals subjected to traumatic brain injury (TBI), enhanced neuroprotection and suppressed neuroinflammation, resulting in improved cognitive and behavioral activities (Shapira et al., 2007; Cowper-Smith et al., 2008; Pang et al., 2016; Wang H. et al., 2016; Huang et al., 2017; Xiao et al., 2017). In spinal cord injury models, GSK-3 inhibitors increased the survival of mature neurons, and promoted axon repair and neuron regeneration (Tuncdemir et al., 2013; Zhang et al., 2016; Lei et al., 2019). In stroke models, GSK-3 inhibition increased neurogenesis and restored function (Venna et al., 2015; Wang W. et al., 2016; Wang L. et al., 2017; Zhao et al., 2017). Glycogen synthase kinase-3 inhibitors were also reported to decrease neuropathic pain (Mazzardo-Martins et al., 2012; Maixner and Weng, 2013; Weng et al., 2014). Finally, studies with GSK-3 inhibitors in epilepsy models revealed attenuation of neurodegeneration (Bhowmik et al., 2015), and a reduction in the rates of seizures (Aourz et al., 2019).

## Conclusion

Over the last decade, the search for GSK-3 inhibitors has been an area of great activity, with a growing number of GSK-3 inhibitors being developed. In addition, the number of relevant indications has dramatically expanded as is well demonstrated in Figure 1. This is reflected in the growing number of clinical trials studying lithium treatment in variety of CNS disorders. Although Phase II clinical trials in AD and PSP with Tideglusib, the only GSK-3 inhibitor to reach the clinic so far, have failed, it is difficult to draw firm conclusions from one molecule, with a specific mode of inhibition (allosteric non-reversible inhibition). In addition, it is possible that the strength of GSK-3 inhibition lies more in improving cognition and behavioral functions in cognitive disorders such as autism and related disease, rather than in protection from neurodegeneration. Similar arguments may be made for lithium with which trials involving neurodegeneration were not successful, while better results were obtained for cognition-related indications. The current appreciation of the importance of personalized medicine, it is possible that treatment with GSK-3 inhibitors should be used only in the fraction of patients who exhibit abnormally high GSK-3 activity. Thus, it may be useful to develop new diagnostic tools in order to identify the patient population who might most benefit from a suitable treatment.

In summary, there is a great promise in using GSK-3 inhibitors in the CNS arena and it is hoped that a large number of recently developed GSK-3 inhibitors will enter the clinic.

## Author Contributions

SA and HE-F wrote the manuscript. HE-F supervised the work. Both authors contributed to the article and approved the submitted version.

## Conflict of Interest

The authors declare that the research was conducted in the absence of any commercial or financial relationships that could be construed as a potential conflict of interest.

## Publisher’s Note

All claims expressed in this article are solely those of the authors and do not necessarily represent those of their affiliated organizations, or those of the publisher, the editors and the reviewers. Any product that may be evaluated in this article, or claim that may be made by its manufacturer, is not guaranteed or endorsed by the publisher.
